# Hematological and Biochemical Characteristics Associated with Cytogenetic Findern Alterations in Adult Patients with Acute Lymphoblastic Leukemia (ALL) from the Northern Region of Brazil

**DOI:** 10.3390/biomedicines12122739

**Published:** 2024-11-29

**Authors:** Dejair da Silva Duarte, Eliel Barbosa Teixeira, Marcelo Braga de Oliveira, Thiago Xavier Carneiro, Lucyana Barbosa Cardoso Leão, Fernando Augusto Rodrigues Mello Júnior, Debora Monteiro Carneiro, Patricia Ferreira Nunes, Amanda Cohen-Paes, Diego Di Felipe Ávila Alcantara, André Salim Khayat, Rommel Mario Rodriguez Burbano

**Affiliations:** 1 Núcleo de Pesquisas em Oncologia, Universidade Federal do Pará, Belém 66073-000, Brazil; dejairduartesilva@gmail.com (D.d.S.D.); elielcn2015@gmail.com (E.B.T.); oliveira.mb23@gmail.com (M.B.d.O.); khayatas@gmail.com (A.S.K.); 2 Hospital Ophir Loyola, Belém 66063-240, Brazil; thiagoxavc@gmail.com (T.X.C.); lucyana_cardoso@yahoo.com.br (L.B.C.L.); fernando.mellojr@hotmail.com (F.A.R.M.J.); deboramonteirocarneiro@gmail.com (D.M.C.); dsnpatriciaferreira@gmail.com (P.F.N.); diegodifelipe10@gmail.com (D.D.F.Á.A.); rommelburbano@gmail.com (R.M.R.B.)

**Keywords:** acute lymphoblastic leukemia, hematological characteristics, biochemical characteristics, chromosome 21 aneuploidy, t(9;22), t(4;11), t(1;19), del(6q), del(9p)

## Abstract

Acute lymphoblastic leukemia (ALL) is an aggressive neoplasm derived from B and/or T cell lineage (B-ALL; T-ALL). For the first time, this study describes, cytogenetically, the karyotypic alterations in adults with ALL in the northern region of Brazil and their relationship with hematological and biochemical characteristics. Through banding analyses, immunophenotyping, as well as hematological and biochemical examination data obtained directly from patients’ records, we found that chromosome 21 aneuploidy was the most frequent. The cytogenetic structural alterations observed with the highest incidence among the patients were: t(9;22), t(4;11), t(1;19), del(6q), and del(9p). In patients presenting with chromosome alterations, we verified that patients with t(4;11) have elevated red blood cell levels and patients with del(9p) presented with distinct and high values of hematological parameters compared to other patients. Regarding biochemical alterations, we observed that patients with translocations (4;11) and del(6q) presented with elevated urea levels compared to other patients, highlighting its relationship to kidney changes and patient prognosis. Thus, our study highlights that variations in hematological and biochemical data are associated with specific cytogenetic changes and other factors, which may impact the prognosis of adult patients with ALL.

## 1. Introduction

Acute lymphoblastic leukemia (ALL) is an aggressive neoplasm derived from B and/or T cell lineage (B-ALL; T-ALL), affecting immature lymphocytic cells in the blood [1]. Adult patients have an incidence of 1 per 100 inhabitants, with the incidence being higher in elderly adults, with a survival decrease related to age advancement [2]. According to the WHO, ALL is characterized mainly by cytogenetic alterations that lead to leukemogenesis [1,3]. Karyotyping is a well-consolidated technique used in these characterizations in adult patients with ALL, as well as fluorescence in situ hybridization (FISH) and reverse transcription polymerase chain reaction (RT-PCR) [4]. Therefore, mapping these changes through genetic and molecular tests is, nowadays, mandatory in the prognosis and risk stratification of patients [5], as well as highlighting the molecular epidemiology of a distinct geographic region. Thus, our study describes, for the first time, cytogenetically, the karyotype alterations in adults with ALL in the northern region of Brazil, and the relationships with hematological, biochemical, and other molecular characteristics in these patients.

## 2. Materials and Methods

### 2.1. Ethical Aspects and Patients

This study was approved by the Ethics Committee of Ophir Loyola Hospital, Belém-Pará (number: 4,409,317). The cohort comprised 45 adult patients of both sexes, being represented by 29 men and 16 females, aged over 18 years (mean 34.71 years, SD = 12,818), diagnosed with acute lymphoblastic leukemia of lineage B and T. Patients were from Ophir Loyola Hospital, a reference hospital in the Para state, Brazil. The exclusion criterion was patients with a suggestive or inconclusive diagnosis for ALL. Furthermore, it is worth noting that it has already been demonstrated that the profile of the Brazilian Amazon populations is distinct from other populations in Africa, Europe, the Americas, and South and East Asia, as it is formed through a high degree of miscegenation [6].

### 2.2. Cytogenetic Characterization

Blood and marrow samples were collected in heparin tubes and transferred to tubes with MarrowMAX^TM^ from Gibco® (Thermo, Grand Island, NY, USA). Samples were collected after 24 h of culture and 0.1 mL of colchicine was added 2 h after each collection. They were subsequently centrifuged (1000 rpm for 10 min), the supernatant was removed, and hypotonization treatment started with potassium chloride (KCl) at a concentration of 0.075 M at 37 °C for 20 min. Samples were centrifuged and fixed three times with methanol/acetic acid (3:1). Cell suspensions were placed on histological slides, and then the GTG banding technique was performed [7,8]. Chromosome classification followed the standards of the International Human Cytogenetic Nomenclature System [9]. Twenty metaphases from each of the patients were analyzed. Such data are available in Supplementary Table S1.

### 2.3. Clinical Data and Biochemical and Hematological Exams

The hematological and biochemical parameters evaluated in this study were collected directly from the medical records of patients with a confirmed diagnosis of ALL, through immunophenotyping. These parameters were evaluated and are available in Table 1. As well as parameters such as sex, lineage, occurrence of death, symptoms presented, and clinical risk were obtained from medical record data.

### 2.4. Symptom Classification

For this analysis, patients were classified into two groups according to the initial symptoms reported in their medical records when they were seen at the Ophir Loyola Hospital. The non-hematological group consisted of patients who reported symptoms such as asthenia, bone pain, weakness, and abdominal pain, while the hematological group had initial symptoms such as anemia, thrombocythemia, fever, and lymphomegaly. Also, we checked whether clinical symptoms were associated with hematological and biochemical changes.

### 2.5. Statistical Analysis

Calculations were performed using the Chicago software, SPSS Inc. (SPSS Inc., released 2008, SPSS Statistics for Windows, Version 17.0). Analyses were performed using Levene’s test for distribution analyses and Student’s *t* tests. Group data are expressed as mean ± standard deviation (SD). Nominal values were analyzed with the Fisher’s exact test and Odds Ratio. Values of *p* ≤ 0.05 were considered significant.

## 3. Results

### 3.1. Cytogenetic

**Figure 1:**
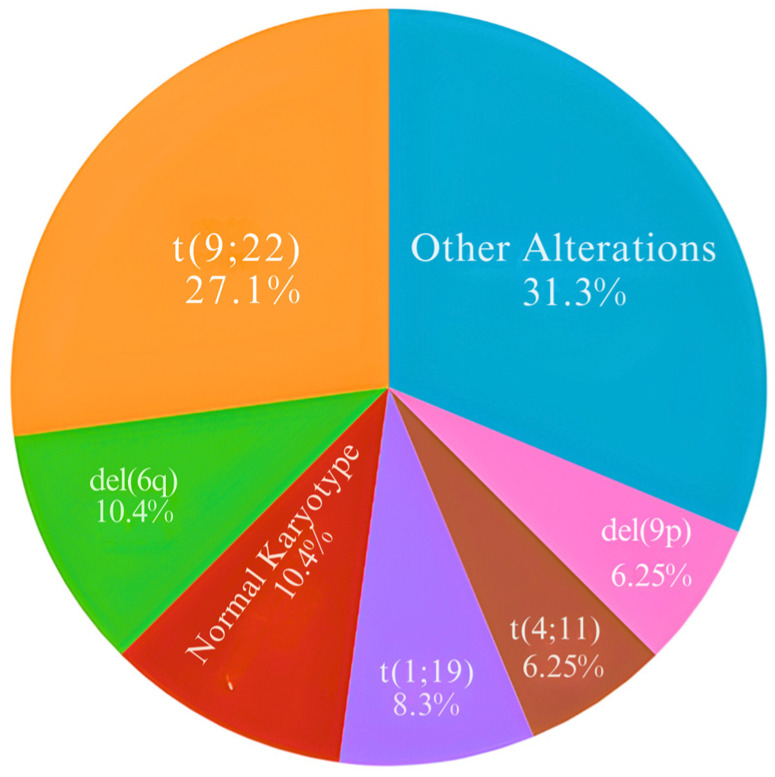
Percentage of cytogenetic alterations and normal karyotype in 46 adult patients with ALL. The other alterations are described in Supplementary Table S1.

The cytogenetic structural alterations observed with the highest incidence among the patients were: t(9;22), t(4;11), t(1;19), del(6q), and del(9p). However, of the 45 karyotypes evaluated, 6 had a normal karyotype (Figure 1). 

**Figure 2:**
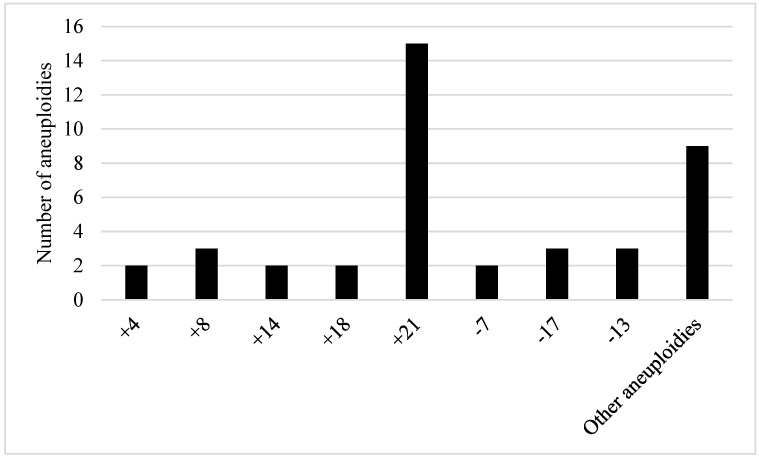
Number of aneuploidies described in 46 patients with ALL.

Among the aneuploidies observed in this study, chromosome 21 was the most frequent aneuploidy among patients with ALL. Also, monosomies were found involving chromosomes −7, −13 and −17. Triploidies of chromosomes +4, +8, +14, +18 were observed too (Figure 2).

### 3.2. Cytogenetic Alterations and Hematological Data

The study evaluated the hematological parameters of adult patients diagnosed with ALL. It was observed that patients who presented the t(4;11) chromosomal translocation had a lower number of red blood cells when compared to other translocations, with mean values of 2.16 (SD = 0.310) and with a *p*-value of 0.047. Patients who had del9p had lower hemoglobin levels (mean = 11.766 g/dL, SD = 2.395) and hematocrit levels (mean = 35.666%, SD = 7.115) compared to the other group with chromosomal abnormalities, with *p* values of 0.035 and 0.035, respectively. However, the mean corpuscular volume (MCV) was higher, with a mean difference of 13.242 fL compared with the other patients with ALL (*p* = 0.005; SD = 6.097). The mean corpuscular hemoglobin (HCM) was also higher with a difference of 4.246 pg vs. the other patients (*p* = 0.001; SD = 2.06). All results are shown in Table 2. 

Patients with ALL and normal karyotype present with smaller amounts of lymphocytes compared to other patients, with a mean value of 1950 m/mm^3^ (*p* = 0.001; SD = 1.56). The mean platelet values, with a mean value equal to 48.571, are lower compared to the other patients with ALL (*p* = 0.001; SD = 43.181). Among the patients evaluated, 26 were male and 16 were female. Male ALL patients had a higher amount of red blood cells (*p* = 0.022; SD = 0.732), hemoglobin (*p* = 0.049; SD = 2.517), and hematocrit (*p*= 0.018; SD = 7.367), with significant mean differences in relation to women (Table 2). 

### 3.3. Cytogenetic Alterations and Biochemical Data

Our study also evaluated changes in biochemical parameters in adult patients with ALL. The patients with ALL with t(4;11) translocations had high mean values of urea, when compared to other patients with ALL; mean equal to 145.66 mg/dL (*p* = 0.005; SD = 53.758). Also, patients with ALL and del6q showed high mean values of urea in relation to the patients evaluated in this study, having a mean value of 109 mg/dL (*p*= 0.031; SD = 116.404). Furthermore, the amount of glucose observed in patients with ALL and initial hematologic symptoms was higher compared to other patients. In addition, the amount of glucose observed in patients with ALL and initial symptoms (non-hematological) was lower than other patients, with a mean equal to 101,875 mg/dL (*p* = 0.027; SD = 25.723). Additionally, male patients with ALL had an elevated amount of creatinine compared to females, with a mean difference of 6686 mg/dL (*p* = 0.041; SD = 12.43). All data are available in Table 3.

## 4. Discussion

### 4.1. Cytogenetic Abnormalities

Our study characterizes cytogenetic changes in the abnormal karyotype in adult patients in the northern region of Brazil and demonstrates how such karyotypes and other factors can be associated with changes in hematological and biochemical values. Notably, karyotyping promotes the detection of recurrent chromosomal rearrangements, being a tool used in the management and risk stratification of adult patients with ALL, in addition to immunophenotyping and molecular diagnosis [1,3]. 

In the present study, chromosome 21 trisomy was the most common aneuploidy in patients with ALL (Figure 2). These phenomena were observed even in of high hyperdiploid and hypodiploid cases and also in almost all haploid cases of ALL [10]. The relationship between chromosome 21 and ALL can be reinforced by the fact that individuals with Down syndrome have a greater risk of developing B-ALL [11,12]. Furthermore, patients with iAMP21-ALL, an amplification in this chromosome, are classified as high risk and at risk of adverse responses to intensive chemotherapy [13,14].

The t(9;22) translocation was the most common structural alteration among patients with ALL, present in 27.1% of the patients in this study (Figure 1). Therefore, the prevalence of *BCR::ABL* in other countries presents a percentual mean of 28% (SD = 7%) and increases with the age of patients with ALL [14,15,16,17,18,19,20,21]. The Ph (Philadelphia) chromosome is formed from a translocation between chromosomes 9 and 22, generating a fusion gene involving the 5′ region of *BCR* fused to the 3′ sequences of *ABL1* [22], a well-established modification in oncohematology. This finding directly points to the treatment strategy, for example, the addition of *BCR::ABL1* tyrosine kinase inhibitors (TKIs) to intensive chemotherapy has significantly improved outcomes for patients with Philadelphia chromosome-positive (Ph+) ALL. Recently, chemotherapy-free regimens with blinatumomab and TKIs have shown excellent results at the forefront and may signal an emerging paradigm shift in the management of Ph+ ALL [23,24,25]. In fact, the use of TKIs in treatment has significantly increased the rates of complete remission of ALL. Thus, we highlight the importance of t(9;22) in the diagnosis by cytogenetic tests in managing patients with ALL. 

We observed that the t(1;19) translocation is present in 8.33% of patients with ALL. The *TCF3::PBX1* gene is the third most prevalent recurrent chromosomal translocation and, worldwide, presents a percentual mean of 6% (SD = 3.1%) [14,21,26,27,28,29]. Such a translocation results in a hybrid protein as a consequence of *TCF3::PBX1* fusion. These differences in outcome between patients who were positive and negative for the *TCF3::PBX1* gene have been described in ALL [30,31,32,33]. Given that studies associate it with a good prognosis, depending on the age group [29,34], patients may also have an intermediate prognosis [35,36] and an unfavorable prognosis [37,38].

In our study, 6.25% of the patients evaluated presented with the t(4;11) translocation. The t(4;11)/*KMT2A::AFF1* translocation is present at a percentage mean of 5.1% (SD = 2.41) of non-Philadelphia adult cases with B cell precursors [18,20,21,27,28,39]. Such translocations occur in B-type precursor cells and is characterized by a higher leukocyte count compared to other ALL subtypes and an immature phenotype, in most cases with a lack of CD10 expression in leukemic cells [39,40]. It is associated with a poor prognosis for ALL patients, as well as a higher risk of relapse [41,42,43]. Thus, the role of alloHSCT in the treatment of t(4;11) BCP-ALL is corroborated. Having an impact on the pre-transplant MRD status, this highlights the need for additional therapeutic intervention and prospective clinical trials focused on this patient population, with the aim of reducing the tumor burden before transplantation and decreasing the risk of relapse after alloHSCT [44].

Furthermore, we observed del(9p) in 6.25% of ALL patients. This alteration was observed in about 9% of cases of acute lymphoblastic leukemia in adults [45]. An important target of deletion of the chromosomal region 9p is the *CDKN2A*, a gene that encodes p16 (*INK4a*) and p14 (*ARF*). Plus, contiguous genes such as *CDKN2B*, which encodes p15 (*INK4b*), or *MTAP*, which encodes methylthioadenosine phosphorylase, can be included in the deletions, and such deletions are also strongly correlated with changes in *PAX5* genes [46,47]. The deleted *CDKN2* gene was frequently observed throughout ALL progression and is considered an unfavorable prognostic marker in long-term outcomes [48]. Studies also show that the *CDKN2* deletion is frequently acquired during the progression of Ph-positive ALL (Philadelphia positive), serving as a poor prognostic marker of long-term outcome in patients with Ph-positive ALL with *CDKN2A* deletion, even after treatment with second-generation tyrosine kinase inhibitors [49].

The del(6q) cytogenetic alteration was present in 10.4% of patients with ALL. They are a recurrent cytogenetic alteration in ALL. The frequency of del(6p) was found to be in the described proportion of 10.1% patients with ALL [50]. This corroborates several studies using *FISH* and a loss of heterozygosity (LOH), which demonstrated several common regions of deletion involving 6q21–q23 [51]. Furthermore, 6q deletions are associated with an intermediate or poor prognostic outcome in adults [52]. The deletion of the long arm of chromosome 6 in ALL patients typically occurs in the region of the *GRIK2* gene most often affected by 6q16 deletions; complete or partial loss of *GRIK2* function may contribute to some of the lymphoid leukemia proliferations [53,54]. In the case of del6q in T-ALL, it was described through genomic analysis and functional models that co-deletion of two contiguous genes on 6q14 increases malignancy through dysregulation of the ribosome–mitochondria axis, suggesting the potential for therapeutic intervention [55].

Among the ALL patients, 10.4% did not present any cytogenetic alterations. This profile is associated with an intermediate prognosis [56]. Normal karyotypes have been observed in approximately 22% of acute lymphoblastic leukemia cases in adults and children [39]. ALL patients with unaltered karyotypes could receive the same prognostic evaluation as ALL patients with *BCR::ABL1*+. For a better prognosis of these patients, allogeneic hematopoietic stem cell transplantation (alloHSCT) should be actively performed [57]. 

### 4.2. Hematological Modifications

**Figure 3:**
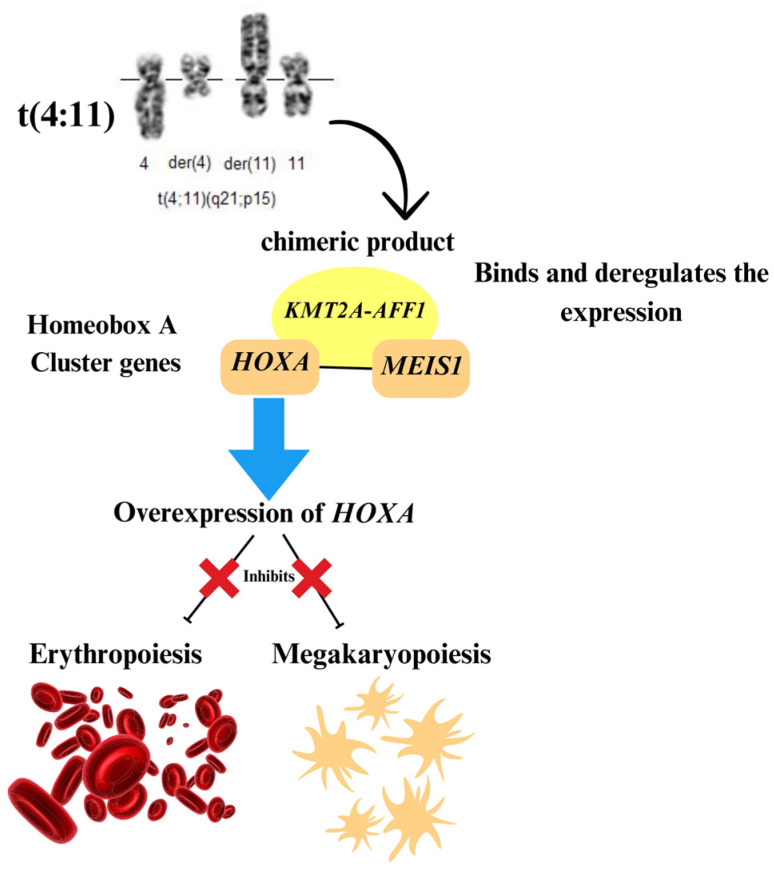
Schematic representation of the biomechanisms related to t(4;11) and the reduction of hematological cells. The image of the cytogenetic translocation was obtained from the *Atlas of Genetics and Cytogenetics in Oncology and Haematology* [62].

Our study observed that patients with ALL and t(4;11) showed significant differences in the count of red blood cells compared to ALL patients with other cytogenetic changes and with a normal karyotype (*p* = 0.47) (Table 2). The chimeric product *MLL-AF4* or *KMT2A/AFF1* binds and deregulates the expression of essential target genes involved in lymphocyte differentiation, including the homeobox A cluster genes *HOXA* and *MEIS1* [58]. Overexpression of *HOXA* inhibits erythropoiesis and megakaryopoiesis, resulting in decreased hemoglobin levels and platelet counts (Figure 3) [59]. The patients in the present study had red blood cell levels below the normal level in healthy adults [60]. Further, Zhang and his collaborators described acute myeloid leukemia patients presenting with t(4;11) had the simultaneous occurrence of anemia and thrombopenia [61], pointing to the influence of this genetic alteration on red blood cell count. 

Patients with ALL and del(9p) showed significant differences in terms of mean hemoglobin (*p* = 0.035), hematocrit (*p* = 0.035), mean corpuscular volume (MCV) (*p* = 0.005), and mean corpuscular hemoglobin (MCH) values (*p* = 0.001), compared to other patients with ALL. The del(9p)-associated loss of the *CDKN2A/B* gene may directly contribute to leukemogenesis [63,64]. There is a report of a patient with ALL and del(9p), where the patient did not respond to chemotherapy treatment and died within one week of induction of chemotherapy (HyperCVAD-A), presenting with symptoms of anemia and thrombopenia [65]. Furthermore, patients with AML and del9p, with a loss of the *CDKN2A* gene, presented with hemoglobin values like those observed in our study [66]. Anemia is also classified according to the MCV value, as microcytic (decreased MCV), normocytic (normal MCV) or macrocytic (increased MCV) [67,68]. The combination of MCV and red blood cell distribution allows for further sub-classification of hematological diseases [69]. This fact highlights the importance of monitoring these parameters in leukemia patients with del(9p).

We observed that patients with ALL and a normal karyotype showed a significant reduction in the average number of lymphocytes and platelets compared to other patients with ALL (*p* = 0.001). Karyotypically normal patients have presented lymphocyte and platelet levels lower than the standards of healthy individuals [60]. It is important to note that, traditionally, white blood cell count, age, and sex are used to this day in patient risk stratification [70,71]. Therefore, these patients are associated with an unfavorable prognosis throughout treatment [57].

In the present study, female patients with ALL showed a significant reduction in the number of red blood cells (*p* = 0.022), hemoglobin (*p* = 0.049), and hematocrit (*p* = 0.018) compared to male patients (Table 1); however, both sexes of patients with ALL had mean amounts of red blood cells, hemoglobin, and hematocrit below the limits considered normal in both sexes [60]. Nevertheless, studies in patients with AML have observed an association between increasing age and being male and a decrease in red blood cell count [72]. It should be noted that changes in red blood cells are related to the development of anemia [68].

### 4.3. Biochemical Alterations

**Figure 4:**
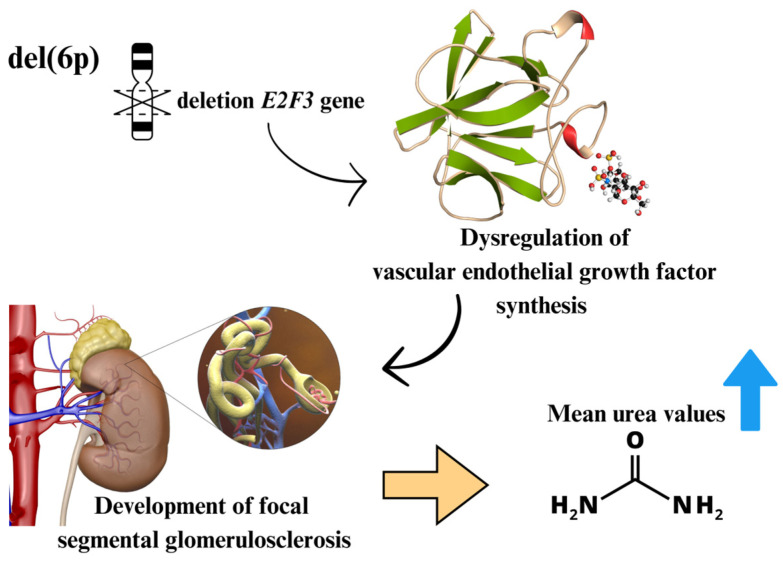
Schematic representation of the biomechanisms related to del(6p) and the increase in urea levels.

Patients with ALL with cytogenetic alterations of the del(6q) and t(4;11) type had high mean urea values compared to other patients with ALL (Table 3). Studies show that del(6p) is associated with the development of focal segmental glomerulosclerosis causing dysregulation of VEGF (vascular endothelial growth factor) synthesis caused by the deletion of the *E2F3* gene (Figure 4) [73]. In both cytogenetic alterations, the amount of urea is above the values considered standard in biochemical tests [74]. The amount of urea is used to assess kidney function, in addition to the body’s nitrogen balance being controlled by regulating urea generation [75]. Acute renal failure has already been observed in patients with ALL at the time of diagnosis [76,77], where the incidence rate of renal failure in patients with untreated ALL ranges from 13% to 25% [78,79]. In this way, renal infiltration is related to a poor prognosis in patients with ALL [80]. However, we highlight the need for more studies related to t(4:11) and urea levels, since we did not observe studies associating both.

Patients with ALL who presented with initial hematological symptoms such as anemia and thrombocytopenia, showed increased glucose levels with significant differences in relation to other patients who did not present these characteristics. In leukemia, oncogenic driver genes such as *MYC* and *RAS* alter metabolic pathways. In ALL, *MYC* leads to increased glucose uptake and glycolytic activity, glutaminolysis, and lipid synthesis [81,82,83,84]. Changes in anabolic metabolism through *RAS* mutations increase glucose uptake and the expression of glycolytic enzymes [85,86]. Such alterations in these oncogenes could explain the high glucose levels observed in the ALL patients evaluated. We observed significant differences between the sexes of patients in relation to creatinine values (*p =* 0.041). In both sexes, patients had creatinine values below what is considered normal when compared to standard values [73]. Patients with ALL and alterations in creatinine levels have already been reported, mainly presenting kidney damage caused by hematological diseases [78,87].

## 5. Conclusions

Our study is the first to describe cytogenetic changes and karyotypes in adult patients with acute lymphoblastic leukemia (ALL) in this admixture adult population in the north of Brazil. We observed that the t(1;19) alteration is described at a higher frequency compared to the world mean. Furthermore, we noted that cytogenetic changes, sex, and initial symptoms of the disease may be linked to variations in hematological and biochemical characteristics in patients with ALL. Regarding changes, we mainly observed that the 6q deletion can lead to kidney damage. Therefore, hematological and biochemical changes associated with cytogenetic findings can help predict prognostic aspects of adult patients with ALL.

## Figures and Tables

**Table 1:** Hematological and biochemical clinical parameters.

Hematological Parameters	Biochemical Parameters
Red cells (million/mm^3^)	Urea (mg/dL)
Hemoglobin (g/dL)	Transaminase (AST)/(U/L)
Hematocrit (%)	Creatine (mg/dL)
Mean corpuscular volume MCV (fL)	Glucose (mg/dL)
Mean corpuscular hemoglobin (pg)	Magnesium (mg/dL)
Mean corpuscular hemoglobin concentration (g/dL)	Potassium (mEq/L)
RDW (%)	Sodium (mEq/L)
Leukocytes (mm^3^)	
Lymphocytes (relative value)/%	
Lymphocytes (absolute value)/(mm^3^)	
Monocyte (relative value)/%	
Monocyte (absolute value)/(uL)	
Neutrophil (relative value)/(%)	
Neutrophil (absolute value)/(mm^3^)	
Eosinophil (relative value)/%	
Eosinophil (absolute value)/(µL)	
Basophils (relative value)/%	
Basophils (absolute value)/(µL)	
Rods (relative value)/(%)	
Rods (absolute value)/(mL/µL)	
Platelets (mm^3^)	

**Table 2:** Hematological clinical exam data.

	Hematological Parameters	Categories	Mean	Min. Value	Max. Value	SD	*p*-Value
**t(4;11)**	Red cells (millions/mm^3^)	t(4;11)	2.163	1.86	2.48	0.310	0.047
		Other	3.081	1.02	4.93	0.765	
**del(9p)**	Hemoglobin (g/dL)	del9p	11.766	7.7	15.4	3.955	0.035
		Other	8.597	5.3	15.6	2.333	
	Hematocrit (%)	del9p	35.666	23.7	44.9	10.861	0.035
		Other	26.133	8.7	45.8	7.115	
	Mean corpuscular volume MCV (fL)	del9p	100.73	95.18	107.26	6.097	0.005
		Other	87.494	74.01	104.65	7.481	
	Mean corpuscular hemoglobin (pg)	del9p	33.030	30.92	34.66	1.915	0.001
		Other	28.783	23.5	34.66	2.061	
**Normal karyotype**	Lymphocytes (mm^3^)	Normal karyotype	1.950	113.4	1185.8	1.560	0.001
		Other	8.003	324.8	5901.3	8.263	
	Platelets (mm^3^)	Normal karyotype	48.571	12	130	43.181	0.001
		Other	142.78	4	396	125.44	
**Sex**	Red cells (millions/mm^3^)	Male	3.214	1.89	4.93	0.7432	0.022
		Female	2.666	1.02	3.58	0.7322	
	Hemoglobin (g/dL)	Male	9.358	5.3	15.6	2.517	0.049
		Female	7.812	2.9	11.6	2.317	
	Hematocrit (%)	Male	28.737	15.7	45.8	7.367	0.018
		Female	23.200	8.7	33.7	6.975	

The values g/dL = grams per liter; mm^3^ = cubic millimeter; Fl = fentoliter; pg = picograms.

**Table 3:** Biochemical clinical exam data.

	Parameters	Categories	Mean	Min. Value	Max. Value	SD	*p*-Value
**t(4;11)**	Urea (mg/dL)	t(4;11)	145.66	80	232	78.08	0.005
		Others	48.57	15	294	53.79	
**del(6q)**	Urea (mg/dL)	del(6q)	109	19	294	116.4	0.031
		Others	48.3	13	157	47.09	
**Initial** **symptoms**	Glucose (mg/dL)	Non-hematological	101.87	69	164	25.72	0.027
		Hematological	140.4	101	207	47.17	
**Sex**	Creatine (mg/dL)	Male	90.55	0.54	6.23	124.3	0.041
		Female	23.69	0.3	2.4	32.8	

The values mg/dL = Milligrams per deciliter.

## Data Availability

The results can be found directly in Supplementary Table S1.

## Supplementary Materials

The following supporting information can be downloaded at: https://www.mdpi.com/article/10.3390/biomedicines12122739/s1.
